# Gender differences in resume language and gender gaps in salary expectations

**DOI:** 10.1098/rsif.2024.0784

**Published:** 2025-06-04

**Authors:** Qian Qu, Quan-Hui Liu, Jian Gao, Shudong Huang, Wentao Feng, Zhongtao Yue, Xin Lu, Tao Zhou, Jiancheng Lv

**Affiliations:** ^1^ College of Computer Science, Sichuan University, Chengdu, People’s Republic of China; ^2^ College of Information and Communication, National University of Defense Technology, Wuhan, People’s Republic of China; ^3^ Faculty of Social Sciences, The University of Hong Kong, Hong Kong SAR, People’s Republic of China; ^4^ CompleX Lab, University of Electronic Science and Technology of China, Chengdu, People’s Republic of China; ^5^ College of Systems Engineering, National University of Defense Technology, Changsha, People’s Republic of China

**Keywords:** computational social science, neural network model, gender gap, written language, resume

## Abstract

How men and women present themselves in their resumes may affect their opportunity in job seeking. To investigate gender differences in resume writing and how they are associated with gender gaps in the labour market, we analysed 6.9 million resumes of Chinese job applicants in this study. Results reveal substantial gender resume differences, where women and men show distinct patterns in both simple language features and high-level semantic structures in the word embedding space of resumes. In particular, women tend to use shorter resumes, longer sentences and a more diverse set of unique words. Neural network models trained on resumes can predict gender with 80% accuracy, and the accuracy decreases with education levels and text standardization requirements. Moreover, while better language skills are associated with higher salary expectations, this positive relationship is magnified for men but weakened for women in women-dominated occupations. This study presents a new venue for the understanding of gender differences and provides empirical findings on how men and women are different in self-portraying and job seeking.

## 1. Introduction

Gender disparities in the labour market have been widely documented, from recruiting new employees to evaluating, promoting and rewarding incumbents [1–4]. Extensive literature provides evidence of gender gaps whereby women are offered fewer opportunities than men during the job search and career development [5–7]. For example, in hiring, women receive fewer callbacks than men for interviews even though they have equivalent resumes [8], and female students are rated as less competent and hireable than male students with identical resumes [9]. Many of these gender gaps are attributed to stereotypes and discrimination [10–12]. Resumes are often the first medium through which employers evaluate job applicants [13], and most studies use names on resumes to indicate applicants’ gender [14]. Presumably, reducing gender information by anonymizing resumes would reduce gender bias in initial resume screening [15,16]. However, some studies provide paradoxical evidence, suggesting that employers may use other implicit signals to infer gender from resumes [17,18]. For example, women using less feminine language in cover letters are less likely to be hired for male-dominated jobs [19].

Among the many clues, written language in resumes provides the most relevant and rich information for gender inference during a job search. Literature from fields as diverse as linguistics, psychology and biology suggests that women and men have different language skills and styles in writing and communication [20–22]. For example, women are less likely to self-promote and use self-promotional language [23]. There are also gender differences in the use of positive and generic words [24], linguistic abstraction [25], lexical and syntactic features [26], readability [27] and gendered language [28,29]. Moreover, women are believed to have better language ability than men during early childhood and before formal education [30,31]. For example, girls have a slight advantage in language acquisition [32], and women score higher in writing achievement [33]. This suggests that gender is, to a large extent, encoded in languages.

Indeed, artificial intelligence (AI) algorithms have demonstrated the capability to infer gender from names and written languages [34–36]. Recently, AI systems have been increasingly used to automate many hiring processes, including resume screening, which reduces cost and improves efficiency [37,38]. At the same time, however, they are found to show bias against some populations, for example, women [39], which causes concerns about fairness and ethics in algorithmic hiring [40]. As the adoption of AI systems continues to shape many hiring processes, especially with the rapid development of deep neural networks and large language models [41,42], AI algorithms, if without human guidance, may discriminate against some populations due to the biases learned from training data and the personal information inferred from resumes [43,44]. Therefore, it is important to understand the extent to which AI algorithms can predict gender from the resumes of job applicants.

Besides gender gaps in hiring, literature also provides evidence showing a gender gap in salary, where women are found to be paid less than men [45–47]. The reasons link to many social factors such as the motherhood penalty and discrimination based on gender [48]. Most of the literature, however, focuses on actual salary, which reflects internal factors (e.g. self-perception and job expectations) and outside factors (e.g. discrimination from employers). Some studies show that women are underpaid because they have lower aspirations [49], and they ask for less salary and engage less in negotiations for a high salary [50–52]. Due to the lack of data on self-assessment and expectations, studies are limited to using actual salary. Resume data containing expected salary allows us to isolate the effect of internal factors.

In this study, we analyse large-scale data covering 6.9 million resumes of Chinese job applicants who disclosed their gender and salary expectations and provided a summary text of their working experiences. We find gender resume language differences whereby women and men show distinct patterns in simple language features and high-level semantic structure in the word embedding space of resumes. We then evaluate the extent to which AI models can predict gender from resumes. We also explore the heterogeneity in gender prediction accuracy across individuals’ education levels and text standardization requirements, comparing resumes with microblogs and scientific publications. Finally, we study how gender and job characteristics affect the benefits of better language skills in salary expectations. Our study provides insights into gender gaps through the lens of resume language.

## 2. Methods

### 2.1. Data

The digital resume data of Chinese job applicants were originally collected from recruitment websites in 2014−2015 (electronic supplementary material, appendix S1). From each resume, we first extracted gender, age, academic degree, current occupation, province of working, expected salary range and a summary text of working experiences. We then standardized academic degrees into four categories (‘secondary and below’, ‘college’, ‘bachelor’ and ‘master and above’), cleaned occupations according to the list provided by the ‘Dictionary of Occupations in China (2015)’ and calculated the expected salary by taking an average for the expected salary range. We dropped resumes that have missing values in either gender or the summary text. This data-cleaning process yielded a sample of 6 917 071 resumes (3 032 622 from women and 3 884 409 from men). The study protocol has been approved by the institutional review board (IRB), and we closely followed the protocol in the study. The resume data were obtained under a data use agreement. All resumes were anonymized during collection and cleaning process (see electronic supplementary material, appendix S1 for details). To measure the economic development status, we further collect each province’s GDP per capita data from the ‘China Statistical Yearbook (2015)’ (electronic supplementary material, appendix S1).

We supplement the analysis of gender prediction on resumes with microblogs and publications, which arguably have different text standardization requirements. The microblog dataset is sourced from Sina Weibo, one of China’s largest social media platforms. It contains tweets posted by 900 000 users (450 094 women and 449 906 men), and gender is self-disclosed in users’ profiles. Microblogs have fewer requirements for text standardization than resumes because people usually share their personal experiences on social media (electronic supplementary material, appendix S1). The publication dataset is sourced from China National Knowledge Infrastructure (CNKI), one of the largest databases of Chinese publications. We collected an initial set of publications, each of which contains an abstract, authors and fields. After inferring the authors’ gender from their names using gender determination application programming interface (API), we only kept publications with no more than two authors of the same gender (electronic supplementary material, appendix S1). This yielded a final sample of 57 184 publications (31 046 by women and 26 138 by men). Publications have more requirements for text standardization than resumes because scientific papers are published in a formalized writing structure and usually after peer review (electronic supplementary material, appendix S1).

### 2.2. Word embedding of resumes

The word embedding representation of resumes was learned using the generative pre-trained transformer (GPT)-based model [53]. Briefly, GPT is a neural network model pre-trained on a large general text dataset to capture the semantic information of the text. Our word embedding is a 768-dimensional vector from the embedding layer of the GPT model. Words (Chinese phrases) from resumes are fed into the GPT model after word tokenization by the neural network model. The output of the embedding layer is the embedding representation of words (electronic supplementary material, appendix S2). Specifically, we first remove stop words from resume summary texts. We then calculate the sentence embedding: ${\text{sent}}_{\text{emb}}=1/m\sum_{i=1}^{m}{\text{emb}}_{\text{i}}\cdot w_{i}$, where m is the number of words in each sentence, ${emb}_{i}$ represents the word embedding of each word and $w_{i}$ represents the weight of each word based on the term frequency–inverse document frequency (if-idf). The vectors of all sentences are summed to obtain the embedding expression of resumes. After learning the representation, we projected the high-dimensional space to a two-dimensional space for visualization using the principal component analysis (PCA) algorithm. The top 50 words most associated with ‘女’ (women) and ‘男’ (men) are identified by the distance in the embedding space, where a smaller distance indicates a stronger association (electronic supplementary material, appendix S3).

### 2.3. Artificial intelligence models for gender prediction

We use two types of AI models to study the predictability of gender in text data, including the summary text in resumes, the tweeting text in microblogs and the abstract text in publications. One is the GPT-based classification model [53], which has two stages in the model’s training. The first stage is pre-training on a very large general text dataset to capture the semantic information of the text, and the second stage is fine-tuning the model on a specific dataset to perform classification tasks. We downloaded the public GPT model from Hugging Face [54] and trained the model on our text data with gender labels. Our GPT-based model contains an embedding layer that maps words or tokens into a 768-dimensional vector representation, and it consists of 12 modified decoder modules in the Transformer that contain the masked multi-head self-attention layer and feed-forward layer. We added two linear layers to perform the gender classification task (electronic supplementary material, appendix S2). The other is the classification model based on the long short-term memory (LSTM) network [55]. The LSTM-based classification model contains an embedding layer that maps words or tokens into a 256-dimensional vector representation, a bidirectional LSTM network layer that contains 512 neurons, an attention layer [56] that helps the model focus on different parts of the input sequence and a softmax layer that suggests the probability of a gender. We trained the LSTM-based model with a back-propagation algorithm and performed an out-sample prediction to test its performance (electronic supplementary material, appendix S2). We present the results from the GPT-based model in the main text and include those from the LSTM-based model as robustness checks in the electronic supplementary material, appendix S4.

### 2.4. Regression model

We explore the association between resume language diversity (LD) and expected salary by employing an ordinary least squares (OLS) regression model that controls for personal, job-related and socioeconomic factors. The empirical specification is given by


(2.1) $$\begin{array}{rl} S_{i}= & \;k_{0}+k_{1}{LD}_{i}+k_{2}{SW}_{i}+k_{3}{LD}_{i}\times {SW}_{i}+k_{4}{Age}_{i}\\ & +k_{5}{GDP}_{i}+k_{6}D^{G_{i}}+k_{7}D^{D_{i}}+\epsilon_{i,j}, \end{array}$$

where the dependent variable $S_{i}$ is the expected salary of individual i in the logarithmic form, and $\epsilon_{i,j}$ is the error term. The independent variable ${LD}_{i}$ is the resume LD measured by the number of unique words per resume, ${SW}_{i}$ is the share of women (SW) in individual i’s occupation, ${LD}_{i}\times {SW}_{i}$ is the interaction term, ${Age}_{i}$ is individual i’s age and ${GDP}_{i}$ is the logged GDP per capita of the province in which individual i works. The two dummy variables are gender $D^{G_{i}}$ and academic degree $D^{D_{i}}$. Details of these variables are provided in the electronic supplementary material, appendix S1, summary statistics of variables in the electronic supplementary material, appendix table S8, and additional regression results in the electronic supplementary material, appendix S5.

## 3. Results

### 3.1. Quantifying gender resume language differences

**Figure 1.**
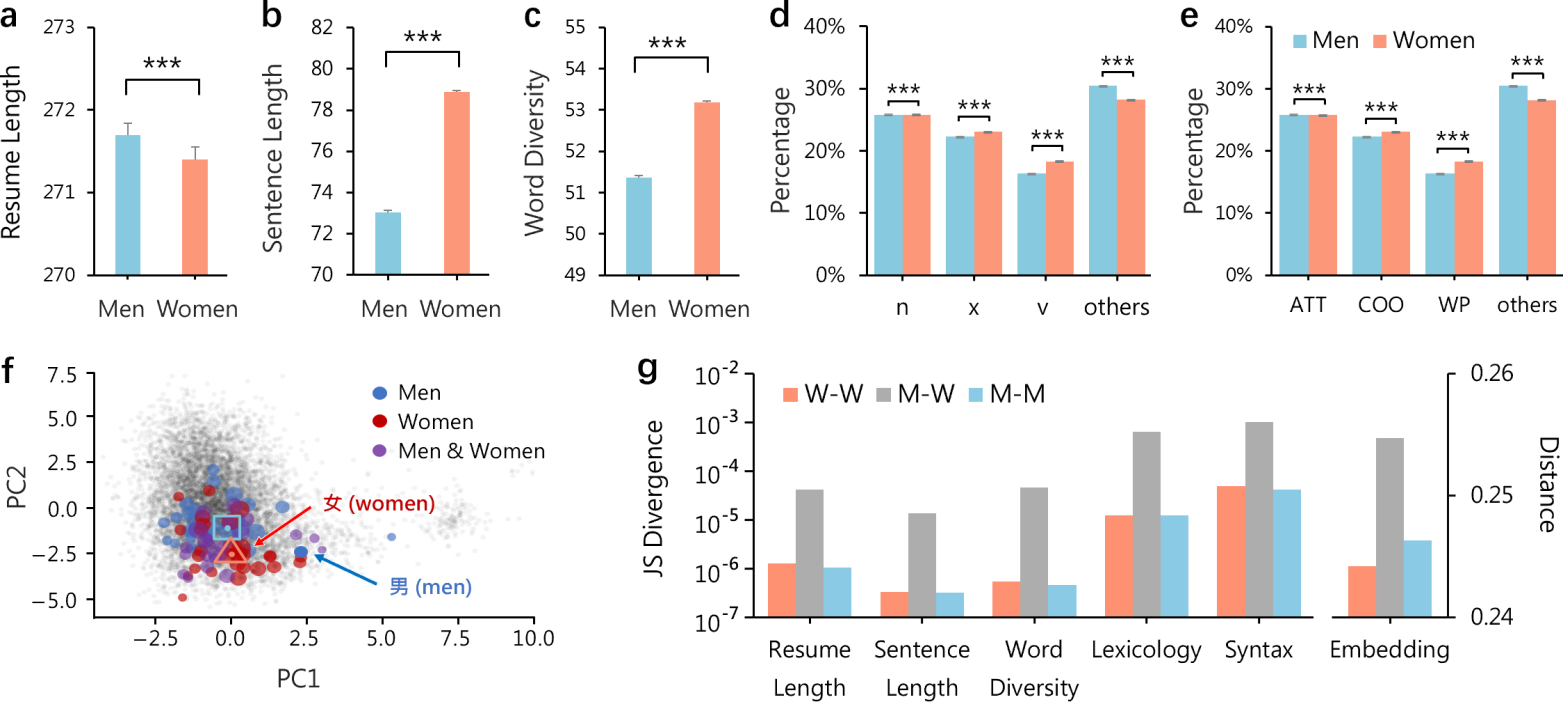
Gender differences in the resume language. (a) The average length of resumes. The total number of Chinese characters measures the length of a resume. Throughout panels, error bars represent standard errors, and the difference between women and men groups is tested using a *t*‐test with significant levels: *p < 0.1, **p < 0.05, ***p < 0.01. (b) The average length of resume sentences. The number of Chinese characters per sentence in a resume measures the sentence length. (c) The average lexical diversity of resumes. The number of unique Chinese words per resume measures the lexical diversity. Chinese words are obtained through tokenization by a language model. (d) The average percentage of lexical categories in resume sentences. The top three categories (n: nouns, x: adjectives and v: verbs) are shown, and all others are grouped together for visualization. (e) The average percentage of syntactic categories in resume sentences. The top three categories (ATT: at attribute of, COO: coordination and WP: punctuation) are shown, and all others are grouped together for visualization. (f) A two-dimensional embedding space of words used in resumes. The top 50 words related to ‘women’ and ‘men’ are shown in red and blue circles, with their geographic centres marked by the hollow triangle and square, respectively. (g) Gender language differences in resumes within and between groups. The JS divergence measures the language difference for each simple language metric, and the cosine distance between the vectors of words used in resumes measures the difference for the embedding space. Details of these measures are shown in §2.

We analyse the resume texts summarizing job applicants’ work experiences and find gender differences in linguistic features and structures of written language. Specifically, while men tend to write more about experiences, presenting a longer resume with more Chinese words (figure 1a), women write longer sentences with an average of about 8% more Chinese words per sentence (figure 1b). This is consistent with literature showing that women have better language skills and use longer English words in their speeches [57]. Indeed, we find that women’s resumes exhibit a higher lexical diversity, measured by the number of unique Chinese words per resume (figure 1c), suggesting that women have a larger vocabulary and use language more expressively [58]. In addition, we find that the resume language structure of women and men exhibits different patterns. Specifically, regarding lexicology (figure 1d), women use more verbs, while men use more gerunds and talk more about locations and quantities. Regarding syntax (figure 1e), women use less attributive structures than men. We also calculated the effect sizes of these features and found that some language characteristics showed substantial differences, while others exhibited smaller differences. Detailed comparisons across genders are shown in electronic supplementary material, appendix S3. These results suggest substantial differences in how women and men write their resumes.

To study high-order semantic features, we learn a representation of resumes using the word embedding technique [11,59], where a neural network model is trained to embed resume words (n-grams) into a high-dimensional space (see §2). The embedding captures word associations and inter-textual coupling semantics [60,61]. In the embedding space, words with a larger association and similarity are closer in distance. We learn the embedding in a 768-dimensional space and employ the PCA algorithm [62] to project it into a two-dimensional space for visualization (see electronic supplementary material, appendix S2 for details). We highlight the top 50 words mostly associated with ‘女’ (women) and ‘男’ (men), respectively, visualization using PCA dimensionality reduction (figure 1f). The t-distributed stochastic neighbour embedding (t-SNE) dimensionality reduction results reveal a similar pattern (electronic supplementary material, appendix S3), suggesting substantial gender differences. Despite the overlap in some common words (about 28%; see electronic supplementary material, appendix table S5), women’s and men’s words occupy different parts of the embedding space, suggesting that they use semantically different words to describe experiences. Specifically, women tend to be associated with ‘艺术’ (art), ‘婴儿’ (infant), ‘教育’ (education) and the largest ordinal such as ‘第一’ (first), while men tend to be associated with ‘汽车’ (car), ‘文件’ (document) and Arabic numerals.

Putting together the five language metrics and the word embedding space, we present a systemic comparison between women and men (figure 1g). Specifically, for each language metric, we calculate the Jensen–Shannon (JS) divergence [63] between the distributions of women (W) and men (M). We find that the JS divergence on comparing cross-gender (M–W) distributions is about two scales larger than that on comparing same-gender (W–W and M–M) distributions (figure 1g, left), suggesting that women and men are substantially different in language metrics. For the embedding space, we calculate the cosine distance between the vectors of words used in women’s and men’s resumes and same-gender resumes. We find that the same pattern continues, where cross-gender differences are larger than same-gender differences (figure 1g, right).

### 3.2. Predicting gender using neural network models

**Figure 2.**
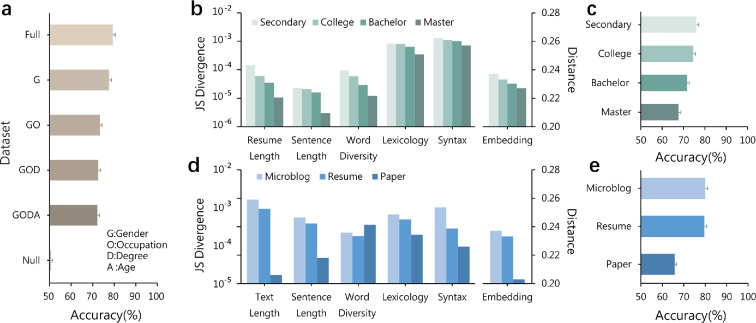
Predicting applicants’ gender from resumes using a neural network model. (a) The model’s gender prediction accuracy on resume datasets. The resume datasets of applicants are matched on gender (G), occupation (O), academic degree (D) and age (A). For instance, G represents a dataset with the same number of women’s and men’s resumes. GO represents a dataset that further ensures the occupation composition of women and men is the same. In the null dataset, gender is randomly shuffled across resumes. (b) A smaller gender difference in resume language for job applicants with a higher academic degree. The gender difference is measured by the JS divergence for the distribution of each resume language metric and by the cosine distance for the embedding space. (c) The gender prediction accuracy for job applicants with different academic degrees. (d) The gender differences in language become smaller as the requirement for text standardization increases from microblogs to resumes to scientific papers. Microblogging text is often short, and its use of words is not standardized. Resume text is more formal and customized by job applicants. Scientific papers are usually peer-reviewed and more standardized than the other two (see electronic supplementary material, appendix S1 for details). (e) The gender prediction accuracy on the three datasets with different text standardization requirements. Throughout panels, the model’s gender prediction results are averaged over 100 realizations, and error bars represent standard errors.

The substantial gender differences in resume language prompt us to study the extent to which job applicants’ gender can be predicted from their resumes. To this end, we train classification neural network models (hereafter, AI models for simplicity; see §2) on the summary texts of job applicants’ working experiences [64]. We find that the model performs well in predicting job applicants’ gender, achieving an accuracy of 80%, much higher than the baseline of 50% (figure 2a). Many factors may affect gender prediction accuracy. As women and men are increasingly balanced in the resume datasets in terms of gender, occupation, academic degree and age (electronic supplementary material, appendix S2), the model’s prediction accuracy gradually decreases to 72%. Still, it remains much higher than the accuracy on a null-model dataset, in which gender is randomly shuffled across all resumes (figure 2a). To ensure the validity of the results, all data samples involving the gender prediction accuracy of classification models have been balanced (electronic supplementary material, appendix S2). These results suggest that job applicants’ gender is, to some extent, encoded in resumes and can be predicted by AI models after controlling demographic and personal characteristics.

The accuracy drop for balanced resume datasets indicates heterogeneity along demographic and personal dimensions. Previous studies found that girls acquire language faster than boys during childhood [65], suggesting that gender language differences are larger before formal education. With improved education, people adopt standardized writing styles with fewer gender-specific features [33]. Consistent with this intuition, we find that gender is less predictable for higher-educated job applicants and from more standardized texts (see §2). Specifically, cross-gender differences in language metrics and word embedding space decrease with the level of education (figure 2b), and the gender prediction accuracy is lower for job applicants with an advanced academic degree (figure 2c).

Similar results are found at the regional level, where a region’s average level of education and the gender prediction accuracy for job applicants from the region exhibit a negative correlation (Pearson’s r=−0.46 and *p* value <0.05; Spearman’s r=−0.34 and *p* value =0.09; see electronic supplementary material, appendix S3). The results suggest that the association is moderate at the regional level and, to some extent, affected by outliers. Moreover, cross-gender differences are larger in microblogging posts and smaller in scientific papers than in resumes (figure 2d), and gender prediction accuracy decreases as the requirement for text standardization increases from microblogs to resumes to scientific papers (figure 2e). These results suggest that improving the level of education and the requirement for text standardization may blur gender-specific language features and shrink the gender resume language differences.

### 3.3. Association with the gender gap in salary

**Figure 3.**
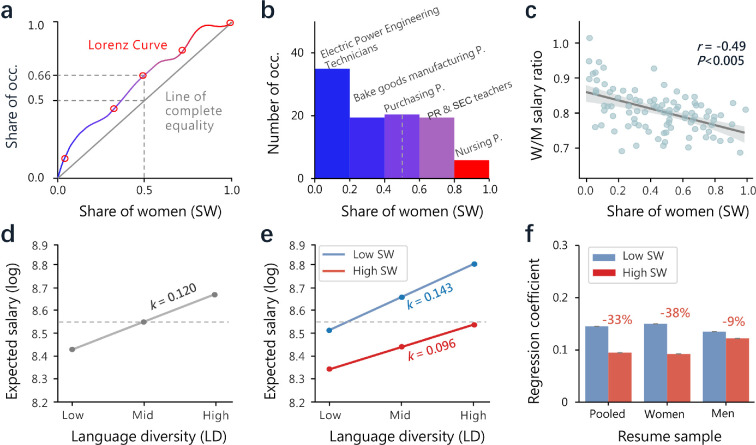
The association between resume language and salary expectations. (a) Unequal gender distribution across occupations. The plot shows the cumulative share of occupations as a function of the SW in an occupation. When the Lorenz curve is a straight line passing through the origin with a slope of 0.5, it indicates that the distribution of women across all occupations is uniform. (b) The histogram of occupations in terms of SW. The *x*-axis represents the SW in occupations, and the *y*-axis indicates the number of occupations within each SW interval. The vertical dashed line marks 0.5, and example occupations are labelled. (c) The negative correlation between SW and women-to-men ratio of expected salary. The Pearson correlation coefficient is denoted as r. A linear fit of data with the 95% confidence interval is shown. (d) Margin plot of the regression on the positive relationship between job applicants’ resume LD and expected salary after controlling individual and job-related factors (see table 1 for details). The ‘low’ and ‘high’ LD in the *x*-axis means 1 s.d. below and above its mean value of 0 (‘mid’), respectively. (e) The positive relationship is moderated by the SW in an occupation. The ‘low’ and ‘high’ SW means 1 s.d. below and above its mean value of 0, respectively. (f) The regression coefficients for analysing women’s, men’s and all resumes. Percentage differences comparing ‘high’ and ‘low’ SW are marked.

The strong predictive power of AI models trained on resumes for job applicants’ gender suggests a potential path that the gender differences in resume language may induce disparities in the labour market. As language skills are more required in some occupations (e.g. registered nurses, teachers and salespeople) than others (e.g. mining machine operators and truck drivers), job applicants with different language skills may have different salary expectations and are offered unequal job opportunities [45,48]. Women generally have better language skills than men, giving them a comparative advantage in jobs that require better language skills. Thus, the share of women (SW) in an occupation, to some extent, indicates the nature of work and its language requirements. Here, we find that the distribution of occupations in terms of SW is uneven, with a Gini coefficient of 0.35 (figure 3a). This suggests the presence of a moderate degree of occupational gender inequality. Many occupations have a very small SW, such as ‘electric power engineering technicians’, while some have a very large SW, such as ‘nursing personnel’. The number of men-dominated occupations is higher than that of women-dominated occupations, with the number of occupations where men comprise 60–100% being 115% greater than the number of occupations where women comprise 60–100% (figure 3b). Moreover, we find a negative correlation (Pearson’s r=−0.49, *p* value <0.01) between SW and the women-to-men expected salary ratio for each occupation (see §2). In other words, women expect fewer salaries in occupations they dominate (figure 3c), which is broadly consistent with previous studies [66,67]. These observations suggest a substantial gender gap in salary expectations of job applicants. We further explore the association between job applicants’ resume language and expected salary. Specifically, we use SW as a proxy for jobs that require better language skills and employ regression models to study the effects of resume LD on expected salary. To investigate the explanatory power of language skills, diversity and other factors on the differences in expected salary more comprehensively, we run the regression on the pooled sample and further built separate models for men and women. We find that the language premium to expected salary is smaller for jobs that require better language skills (table 1 and figure 3). First, the results from an OLS regression model that controls for individual and job-related factors (see §2) suggest that LD has a positive effect on expected salary (model 1 in table 1 and figure 3d). Second, SW has a negative effect on expected salary (model 2 in table 1), and it moderates the relationship between LD and expected salary (model 3 in table 1) such that their positive relationship is less pronounced for jobs with a high SW (figure 3e). In other words, the language salary premium is diminished in women-dominated jobs. Third, the moderating effects of SW for women and men are in the opposite direction (figure 3f). Specifically, when SW is high, the language salary premium is smaller for women (model 4 in table 1) but larger for men (model 5 in table 1). These results suggest that the benefits of better language skills for salary expectations depend on not only the nature of work but also the gender of job applicants. For instance, women with better language skills expect disproportionately lower salaries in women-dominated occupations than in men-dominated occupations (see electronic supplementary material, appendix S5 for detailed results). Similar results were obtained using quantile regression, further supporting this conclusion. Detailed results are provided in electronic supplementary material, appendix S5.

**Table 1.** Results of OLS regressions on the relationship between LD and expected salary of job applicants.

variables	dependent variable: expected salary (log)				
	pooled sample			women	men
	model 1	model 2	model 3	model 4	model 5
LD	0.188^***^	0.121^***^	0.120^***^	0.120^***^	0.126^***^
	(0.000)	(0.000)	(0.000)	(0.000)	(0.000)
SW		− 0.104^***^	− 0.107^***^	− 0.131^***^	− 0.082^***^
		(0.000)	(0.000)	(0.000)	(0.000)
LD × SW			− 0.024^***^	− 0.028^***^	− 0.006^***^
			(0.000)	(0.000)	(0.000)
age		0.167^***^	0.167^***^	0.152^***^	0.176^***^
		(0.000)	(0.000)	(0.000)	(0.000)
GDPpc		0.081^***^	0.081^***^	0.085^***^	0.079^***^
		(0.000)	(0.000)	(0.000)	(0.000)
D (gen.)	no	yes	yes	no	no
D (deg.)	no	yes	yes	yes	yes
adj. $R^{2}$	0.107	0.392	0.394	0.376	0.329
RMSE	0.541	0.446	0.446	0.400	0.477

The observations are 3 518 819 individuals. All independent variables are mean-centred before being included in the OLS regressions except for the three dummy variables: gender, academic degree and occupation. Robust standard errors are reported in parentheses. Significant levels: *p < 0.1, **p < 0.05, ***p < 0.01.

## 4. Discussion

We study gender differences in the written language of resumes in which Chinese job applicants describe their working experiences and disclose their demographics and job-related information. Our analysis reveals gender differences in resume language, where women’s and men’s resumes exhibit distinct patterns in linguistic features and grammar structures. This study analysed a wide range of variables, most of which supported the main conclusions of the study, some heterogeneity was also observed (see electronic supplementary material, appendix S3 for details). In particular, women tend to write longer sentences and show a higher language diversity. The results suggest that women tend to have better language skills, which is consistent with previous studies [33,68]. Moreover, words mostly associated with women and men occupy different areas of the embedding space that capture high-order semantic associations among words. Together, these findings suggest that gender is encoded in the written language of resumes. Indeed, we find that AI models trained on resumes can predict job applicants’ gender with high accuracy. As companies increasingly use AI algorithms to automate resume screening, our findings are alarming because gender can be recovered from the text of resumes. This may increase the concerns about fairness in algorithmic decisions and gender bias in hiring. We find that AI models’ predictive power towards gender decreases with the level of education and the requirement for text standardization. This may suggest possible practices for job applicants to blur gender information in their resumes, such as using more standardized resume templates.

The gender language differences may exert unequal effects on women’s and men’s job expectations. Our analysis shows that, while better language skills are associated with higher salary expectations, the nature of work plays a moderating role. We use the share of women in an occupation as an imperfect proxy for the nature of work and find that the benefits of better language skills are diminished in women-dominated jobs, which may be due to intense competition. When we only consider women, the moderating effect continues and is strengthened, suggesting that the language benefits for women are more diminished in women-dominated jobs. By contrast, when we only consider men, the moderating effect is reverted and weakened, suggesting that the language benefits for men are more magnified in women-dominated jobs. These findings paint a rich picture showing that the impact of language on job expectations depends not only on gender but also on the nature of work. Therefore, women and men may have different job-seeking strategies in terms of whether to self-disclose gender [19]. We showed the robustness of our findings using OLS regression and quantile regression.

Despite efforts to understand gender differences in resume language, our analysis has several limitations, which should be noted when interpreting the results. First, although the resume data are large-scale, their representation of the formal labour market is limited by the enrolment and demographics of job applicants on the recruitment platforms. Second, our analysis focuses only on the written language of job applicants’ resumes in the Chinese labour market. It remains future work to investigate whether the findings can be generalized to other languages, cultural contexts and labour markets, where relevant data are accessible. Third, we use expected salary to measure job applicants’ labour market expectations. It captures self-assessment more directly, which allows us to isolate the effects from the employer side, but it may not represent received salary despite the strong correlation between the two [69,70]. Fourth, the data do not allow us to claim any causality about whether resume language differences cause gender pay gaps or vice versa. Field experiments may be designed and implemented to explore further the mechanisms underlying these observations.

Taken together, our analysis of large-scale resume data reveals gender differences in resume language, and AI models can predict gender from resumes with high accuracy. While better language skills are associated with higher salary expectations, this language benefit is magnified for men but diminished for women in women-dominated jobs. Our results provide insights into gender gaps in the labour market through the lens of resume language. The findings have the potential to help inform policies to improve fairness in hiring in the age of AI, such as by advocating gender-neutral resumes.

## Data Availability

The data and code necessary to replicate the statistical analyses and main figures are available on Zenodo [71]. Anonymized and de-identified resume data are protected by a data use agreement. Those who are interested in the raw data may contact the corresponding authors for access after obtaining IRB approval (see electronic supplementary material, appendix S1). Supplementary material is available online [72].
